# Relationships Between Chemical Defenses of Common Toad (*Bufo bufo*) Tadpoles and Bacterial Community Structure of their Natural Aquatic Habitat

**DOI:** 10.1007/s10886-020-01184-4

**Published:** 2020-05-28

**Authors:** János Ujszegi, Balázs Vajna, Ágnes M. Móricz, Dániel Krüzselyi, Kristóf Korponai, Gergely Krett, Attila Hettyey

**Affiliations:** 1 Lendület Evolutionary Ecology Research Group, Plant Protection Institute, Centre for Agricultural Research, Herman Ottó út 15, Budapest, 1022 Hungary; 2 Department of Microbiology, Eötvös Loránd University, Pázmány Péter sétány 1/C, Budapest, 1117 Hungary; 3 Department of Pathophysiology, Plant Protection Institute, Centre for Agricultural Research, Herman Ottó út 15, Budapest, 1022 Hungary; 4 Danube Research Institute, Centre for Ecological Research, Karolina út 29, Budapest, 1113 Hungary

**Keywords:** Bufadienolide, Local adaptation, Toxin production, *Bufo bufo*, Pond microbiota

## Abstract

Many organisms synthesize secondary metabolites against natural enemies. However, to which environmental factors the production of these metabolites is adjusted to is poorly investigated in animals, especially so in vertebrates. Bufadienolides are steroidal compounds that are present in a wide range of plants and animals and, if present in large quantities, can provide protection against natural enemies, such as pathogens. In a correlative study involving 16 natural populations we investigated how variation in bufadienolide content of larval common toads (*Bufo bufo*) is associated with the bacterial community structure of their aquatic environment. We also evaluated pond size, macrovegetation cover, and the abundance of predators, conspecifics and other larval amphibians. We measured toxin content of tadpoles using HPLC-MS and determined the number of bufadienolide compounds (NBC) and the total quantity of bufadienolides (TBQ). AICc-based model selection revealed strong relationships of NBC and TBQ with bacterial community structure of the aquatic habitat as well as with the presence of conspecific tadpoles. The observed relationships may have arisen due to adaptation to local bacterial communities, phenotypic plasticity, differential biotransformation of toxin compounds by different bacterial communities, or a combination of these processes. Bacterial groups that contribute to among-population variation in toxin content remain to be pinpointed, but our study suggesting that toxin production may be influenced by the bacterial community of the environment represents an important step towards understanding the ecological and evolutionary processes leading to microbiota-mediated variation in skin toxin profiles of aquatic vertebrates.

**Electronic supplementary material:**

The online version of this article (10.1007/s10886-020-01184-4) contains supplementary material, which is available to authorized users.

## Introduction

Many organisms are capable of synthesizing secondary metabolites de novo, which can act as chemical defenses against predators, competitors and pathogens (Brodie 2009; Mebs 2001; Toledo and Jared 1995). Despite a growing body of information about the functions and ecological roles of defensive chemicals in plants and marine invertebrates, information regarding spatial and temporal variation in toxin production, and its relationships with environmental factors has remained limited in vertebrates (Hettyey et al. 2014).

The nature and magnitude of chemical defense generally depend on the relationship between the benefit of the defense and its costs (McCall and Fordyce 2010; Tollrian and Harvell 1999). Intraspecific variation in toxin production among populations can arise at least through three mechanisms: local adaptation, phenotypic plasticity and genetic drift. If local environmental changes are highly predictable, but differ among sites, different levels of constitutive defenses are expected. This results in local adaptation with fixed toxin levels: members of populations are highly toxic if encounter rates with natural enemies are high and less toxic if the threat posed by enemies is relatively low (Hague et al. 2016). In contrast, environments where ecologically important factors vary unpredictably favor the evolution of phenotypic plasticity, which is the ability of individual genotypes to produce different phenotypes in different environmental conditions (DeWitt and Scheiner 2004; Harvell 1990; West-Eberhard 1989). In case of genetic drift, spatial variation in toxin levels can appear among small, genetically isolated populations caused by random processes leading to changes in allele frequencies (Nei et al. 1975).

Steroidal bufadienolides are among the most studied groups of biologically active compounds constituting chemical defenses. They have been isolated from both plant and animal sources and are known to block membrane Na^+^/K^+^-ATPases, making them cardiotoxic if present at sufficient quantities (Daly 1995; Krenn and Kopp 1998; Steyn and van Heerden 1998). Several toad species in the family Bufonidae are known to synthesize bufadienolides de novo in the serous glands of their skin already from early larval stages on (Hayes et al. 2009a; Ujszegi et al. 2017; Üveges et al. 2017). These skin-toxins may be effective against natural enemies including predators (Denton and Beebee 1991; Hantak et al. 2016; Kruse and Stone 1984; Peterson and Blaustein 1991; Shine 2010), competitors (Bókony et al. 2018) and potential pathogens (Barnhart et al. 2017; Cunha Filho et al. 2005; De Medeiros et al. 2019; Tempone et al. 2008). However, little is known about what evolutionary processes and environmental factors influence bufadienolide synthesis.

In a previous study on among-population variation in the toxin content of larval common toads (*Bufo bufo*) in natural habitats we observed that tadpole toxin content was related to the density of conspecifics and to pond permanence, but not to predator abundance (Bókony et al. 2016). However, in that study, microbial communities of the studied ponds were ignored. As demonstrated by Cunha Filho et al. (2005), bufadienolide compounds synthesized in the skin of *Rhinella* (=*Bufo*) *rubescens* have potent antimicrobial activity against both Gram-positive and Gram-negative bacteria. Also, bufadienolides can inhibit the growth of *Batrachochytrium dendrobatidis*, a fungal pathogen associated with global amphibian declines (Barnhart et al. 2017). Although bufadienolides are present, defensive antimicrobial peptides (AMPs) are lacking from the skin of bufonids (Conlon 2011; König et al. 2015). This suggests that bufadienolides play an important role in skin-based immune-defense, and as such, may be highly responsive to changes in the microbial community present in their environment. Finally, it has been demonstrated that some microbes can biotransform bufadienolide compounds (Hayes et al. 2009b), which may in turn also contribute to among-population variation in toxin content.

We investigated whether the composition and quantity of defensive skin toxins in common toad (*Bufo bufo*) larvae may be influenced by the bacterial community present in the environment. To achieve this, we conducted a field survey in 16 natural habitats of the common toad. We related bufadienolide profiles of larval toads to bacterial community structure of their aquatic environment, while controlling for other, potentially influential biotic factors and abiotic pond parameters. We predicted to find a positive relationship between toxin content of tadpoles and the density of their competitors, as well as a strong influence of the bacterial community present in the aquatic environment. We chose to investigate the relationship between the microbial community of the aquatic environment and skin toxin content because it is the water surrounding anuran larvae that serves as the source of the microbiota colonizing their skin. At the same time, microbes present in the immediate environment can have a decisive influence on the skin-based immune-defense of amphibians, such as on the synthesis of AMP-s (Krynak et al. 2016). While it is the microbes that get into direct contact with individuals that matter for the immune-response, the microbial community present on the skin differs from the environmental pool (Rebollar et al. 2016; Walke et al. 2014) and is already selected by skin-secreted chemical defenses (Vartoukian et al. 2010).

## Materials and Methods

*Data collection*. In late May and early June 2015, we visited 16 ponds in the Pilis-Visegrádi Mountains, Hungary, known to be common toad breeding sites (Vági et al. 2013). These ponds are located in deciduous forests between 200 and 570 m above sea level and are known to dry out every few years so that fishes are not present. Surface area ranged between 66 and 3699 m^2^, maximal water depth between 30 and more than 100 cm (Table 1; Electronic supplementary material 1). We estimated canopy cover and pond macrovegetation cover as percentage of pond surface in 5% increments. Average water conductivity and pH were calculated from measurements taken on 5 randomly collected water samples per pond, measured by a portable electrochemistry meter (Consort C 6020 T). The above pond parameters are related to the probability of desiccation and are important parameters that can influence both the development and physiological performance of tadpoles (McDiarmid and Altig 1999), but also the community structure of the aquatic microbiota (Krynak et al. 2015, 2016). We estimated the density of predators, as well as of conspecific and heterospecific tadpoles by performing 1 m long sweeps (ca. 0.4 m^2^) along the bottom of ponds with dip-nets, and subsequent counting of captured animals. We took 4–12 dip-net samples depending on pond size, while taking care to represent microhabitats according to their share of pond area. Subsequently, for each pond we calculated the density of animal taxa as the average number of captured individuals across all dip-net samples. For the analysis of the bacterial communities of ponds, we collected 2 L composite water samples into autoclave-sterilized glass bottles from 10 locations within each pond where tadpoles were present (from water depths ranging between 10 and 45 cm). Water samples were transported on ice to the Department of Microbiology, Eötvös Loránd University, Budapest, Hungary, and stored at 4 °C until further analysis.

**Table 1:** Locations of sampling sites. Sample sizes and average developmental stages (according to Gosner 1960) are also shown. For habitat characteristics of sampling sites please see Electronic supplementary material1

Pond ID	Pond name	Date of visit	Latitude	Longitude	*N* of tadpoles per pond	Mean Gosner stage
1	Alsó-hosszúrét	27.05.2015	47.7155	19.0227	4	35
2	Bükkipuszta	01.06.2015	47.7013	18.9493	10	26.7
3	Felső-hosszúrét 1	08.06.2015	47.7266	19.0158	10	27
4	Felső-hosszúrét 3	08.06.2015	47.7268	19.0167	10	37.2
5	János-tó	27.05.2015	47.7143	19.0197	10	33.5
6	Mélymocsár	03.06.2015	47.7076	19.0401	3	27
7	Nagykovácsi-tó	08.06.2015	47.5764	18.8686	10	36.6
8	Paprét-középső	03.06.2015	47.7389	19.0118	5	27
9	Szárazfarkas-belső	01.06.2015	47.7345	18.8188	10	31.1
10	Szarvasszérű	08.06.2015	47.7294	19.0069	9	26.5
11	Szarvasszérű-megálló	08.06.2015	47.7299	19.0086	10	27.5
12	Sóstó	04.06.2015	47.7753	19.0042	10	34.6
13	Sóstó-zsombékos	04.06.2015	47.7748	19.0044	10	33.6
14	Vörös-dagonya	01.06.2015	47.7062	18.9227	10	36.1
15	Vértes-Észak	03.06.2015	47.7411	19.0439	10	33.7
16	Zánkó	03.06.2015	47.7392	19.0257	9	35

Mean developmental stage (Gosner, 1960) of collected toad tadpoles varied among ponds between 26 ± 1 and 37.2 ± 1.2 (mean ± SD). Common toad tadpoles are known to produce toxins de novo (Üveges et al. 2017) and contain analyzable quantities of toxins at these stages in natural populations (Bókony et al. 2016). We sampled tadpoles by dip netting at several locations within ponds and haphazardly selecting from among the captured specimens ten individuals per pond. We fixed tadpoles in 1 ml absolute HPLC grade methanol, and stored samples at −20 °C.

*Bacterial community analysis based on terminal restriction fragment length* polymorphism. We filtered 700 mL aliquots of each water sample through a 0.45 μm pore-sized cellulose nitrate membrane filter (Millipore, Billerica, MA, USA). Environmental DNA was extracted from the filters using the PowerSoil® DNA Isolation Kit (MoBio Laboratories, Carlsbad, CA, USA) according to the manufacturer’s instructions, with the exception that cell disruption was achieved by shaking at 25 Hz for 2 min using a Mixer Mill MM301 (Retsch, Haan, Germany).

For PCR amplification we used HEX-labelled 27F (5′-AGA GTT TGA TCM TGG CTC AG-3′) and 534R (5′-ATT ACC GGG GCT GCT-3′) 16S rDNA-specific primers (Lane 1991). The PCR mixture contained 2.5 U DreamTaq™ DNA Polymerase (Thermo Fisher Scientific, Waltham, MA, USA), 1× DreamTaq™ Buffer (Thermo Fisher Scientific), 0.2 μL of each dNTP, 0.3 μM of each primer, 20 μg BSA (Thermo Fisher Scientific), and 1 μL of template DNA in a final volume of 50 μL. Thermal profile consisted of an initial denaturation at 98 °C for 5 min, followed by 32 amplification cycles (94 °C for 30 s, 52 °C for 30 s and 72 °C for 30 s), and a final extension step at 72 °C for 10 min. Aliquots of the labelled PCR products (13 μL) were digested in a final volume of 20 μL with 1.5 U restriction endonucleases AluI and Bsh1236I (Thermo Fisher Scientific), separately for 3 h at 37 °C. The purification of enzymatic digests and electrophoresis of labelled fragments were carried out as described previously (Sipos et al. 2007).

*Analysis of bufadienolides*. We homogenized tadpoles with a VWR VDI 12 blender and attached IKA S12N-7S dispersing tool. We dried samples under vacuum at 45 °C using a rotary evaporator (Büchi Rotavapor R-134), and measured dry mass to the nearest 0.1 mg using an analytical balance (Sartorius Entris 224i-1S). We redissolved the dried samples in 1 ml absolute HPLC grade methanol, which was aided by brief exposure to ultrasound in a bath sonicator (Tesla UC005AJ1). Finally, we filtered samples with 0.22 μm pore sized FilterBio nylon syringe filters and stored them at −20 °C until further analysis.

We analyzed bufadienolide compounds using high-performance liquid chromatography coupled with diode-array detector and mass spectrometry (HPLC-DAD-MS) on a Shimadzu LC-MS 2020 instrument (Shimadzu, Kyoto, Japan) that consists of a binary gradient solvent pump, a vacuum degasser, a thermostated autosampler, a column oven, a diode array detector and a single-quadrupole mass analyzer with electrospray ionization (ESI-MS) We identified the chromatographic peaks as bufadienolides based on their UV spectrum (Hayes et al. 2009a) and by comparing their retention time and mass spectrum to commercially available standards of bufalin, bufotalin, resibufogenin, gamabufotalin, areno- and telocinobufagin (Biopurify Phytochemicals, Chengdu, China), cinobufagin (Chembest, Shanghai, China), cinobufotalin (Quality Phytochemicals, New Jersey, USA), digitoxigenin (Santa Cruz Biotechnology, Dallas, TX, USA) and marinobufotoxin (kindly provided by Prof. Rob Capon, Institute for Molecular Bioscience, University of Queensland, Australia). We also compared results to those obtained on a sample we took from an adult male common toad by gently massaging the parotoid glands. As UV spectra are more characteristic in adults (clean and concentrated) than homogenized tadpoles, this helps in detecting unidentified compounds (if molecular standards are absent) comparing retention time and m/z values. Chromatographic separations were carried out at 35 °C on a Kinetex C18 2.6 μm column (100 mm × 3 mm i.d.) in series with a C18 guard column (4 mm × 3 mm i.d.) using 10 μL injections. Eluent A was 5% aqueous acetonitrile with 0.05% formic acid and eluent B was acetonitrile with 0.05% formic acid. The flow rate was 0.8 mL / min and the gradient was as follows: 0–2 min, 10.5–21.1% B; 2–15 min, 21.1–26.3% B; 15–24 min, 26.3–47.4% B; 24–25 min, 47.4–100% B; 25–30 min 100% B; 30–31 min 100–10.5% B; 31–35 min 10.5% B. ESI conditions were as follows: desolvation line (DL) temperature: 250 °C; heat block temperature: 400 °C; drying N2 gas flow: 15 L / min; nebulizer N2 gas flow: 1.5 L / min; positive ionization mode. Data was acquired and processed using the software LabSolutions 5.42v (Shimadzu Corp., Kyoto, Japan).

*Statistical analyzes.* To avoid redundancy by entering closely related predictor variables into statistical analyzes, we first checked for possible correlations between habitat characteristics using non-parametric correlations (Spearman’s rho) and only used variables whose pairwise correlation coefficients did not exceed 0.5. Consequently, we had to ignore water depth, shade, pH and conductivity (Table OR 1 in Electronic supplementary material 2).

TRFLP chromatograms were analyzed with the GeneMapper® Software v3.7 (Applied Biosystems, Foster City, CA, USA). Only TRFs longer than 50 bps were used. Further data processing was carried out according to an updated script of Abdo and colleagues (Abdo et al. 2006; the R script is available upon request). We applied the following parameters: noise filtration based on standard deviation (multiplier = 3) of peak area, TRF alignment with 1 bp clustering threshold. The resulting alignment was compared to the raw chromatograms and corrected manually if necessary. For normalization the relative abundance of each detected TRFs within a given TRFLP profile was calculated. In order to get a more robust result, we combined the data matrix obtained using the AluI and Bsh1236I enzymes.

The bacterial community structure based on the TRFLP data was visualized with nonmetric multi-dimensional scaling (NMDS) with 3 dimensions (stress = 0.0997) using the vegan package (Oksanen et al. 2016) in R (R Development Core Team 2016; http://www.r-project.org/). The 3D NMDS was preferred over the 2D NMDS because the former had a lower stress value and differed more significantly from simulated randomized data matrices generated using the ‘oecosimu’ function in the vegan package.

From three ponds we were only able to collect 5, 4, and 3 tadpoles, and during sample preparation we lost one sample each from 2 further ponds. This resulted in a total of 140 samples on bufadienolide content of tadpoles (Table 1). We described chemical defenses with two variables: we determined the number of bufadienolide compounds (NBC) and calculated total bufadienolide quantity (TBQ) for each animal. We assumed a compound to be present when its signal was at least three-times higher than random noise in the chromatogram (when the area below the curve was larger than 4000). Second, we estimated the quantity of each bufadienolide compound from the area values of chromatogram peaks based on the calibration curve of the bufotalin standard, and summed up these bufotalin-equivalent values to obtain an estimate of total bufadienolide quantity (TBQ) per individual. The use of the calibration curve of the bufotalin standard to obtain approximate estimates of bufadienolide quantities has been successfully used before in similar studies (Bókony et al. 2016; Hagman et al. 2009; Ujszegi et al. 2017). We analyzed NBC with cumulative link mixed modeling procedures (CLMM) with a logit link function and equidistant threshold using the ordinal R-package (Christensen 2015). We entered log_10_-transformed values of TBQ to enhance normality of model residuals and homogeneity of variances and used linear mixed modeling procedures (LMM). We performed model selection relying on Akaike’s information criterion corrected for sample sizes (AICc).

Full models included pond ID as a random factor, and the following covariates as fixed factors: developmental stage of toad tadpoles; the three NMDS axes created from TRFLP data (NMDS 1, 2 and 3) describing the bacterial community of ponds, density of conspecific larvae, density of other amphibian larvae and density of predators (biotic factors), pond surface area and vegetation cover (abiotic pond parameters). As we were predominantly interested in effects of the bacterial community on toxin variables, we created the following models a priori: 1. null model, containing only pond ID as a random factor; 2. pond ID + developmental stage of toad tadpoles; 3. pond ID + developmental stage of toad tadpoles + NMDS axes describing bacterial community; 4. pond ID + developmental stage of toad tadpoles + NMDS axes describing bacterial community + biotic factors; 5. pond ID + developmental stage of toad tadpoles + NMDS axes describing bacterial community + abiotic pond parameters; 6. pond ID + developmental stage of toad tadpoles + NMDS axes describing bacterial community + biotic factors + abiotic pond parameters. We used the maximum likelihood method for estimations and compared models using the MuMIn package (Bartoń 2017) in R. We entered log_10_-transformed values of pond surface, density of conspecific larvae and density of predators. We checked the homogeneity of variances using diagnostic plots. We calculated variance inflation factors (VIF) for each variable to detect multicollinearity, which was not found. Models were considered significantly different from each other in case of difference by more than four AICc values. In case of more than one best-supported model, effects were estimated using model averaged coefficients (MuMin package, conditional average matrix). We also compared models containing all the measured abiotic parameters (regardless of correlations between them) or using the mass corrected TBQ with the same methods described above, which results can be seen in Electronic supplementary material 2. We ran all analyses in R 3.4.0.

## Results

In total, we identified 12 compounds as bufadienolides based on their UV spectra, but only one of these was found to be identical with one of the standards (marinobufotoxin). The number of bufadienolide compounds present in individual tadpoles ranged between 8 and 12 compounds. The frequency of occurrence of some compounds varied largely between ponds (i. e. compound 2, 3, marinobufotoxin, 5 and 10), whereas other compounds were consistently present in most sampled tadpoles in all ponds (Table 2).

**Table 2:** Percentage occurrence (occ. %) of detected bufadienolide compounds (sorted by m/z value) and their mean amount (mean ng) in the sampled tadpoles at given ponds. Mean NBC and TBQ calculated for each pond are also shown. Pond ID accords to the first column in Table 1. Rt: Retention time (min); m/z: mass/charge (M + H^+^)

Pond ID	Compound 1		Compound 2		Compound 3		Marinobufotoxin		Compound 4		Compound 5		Compound 6		Compound 7		Compound 8		Compound 9		Compound 10		Compound 11		Mean NBC per tadpole	Mean TBQ (ng) per tadpole
	Rt: 7.2		Rt: 6.7		Rt: 7.4		Rt: 17.7		Rt: 16.2		Rt: 18.5		Rt: 11.6		Rt: 10.2		Rt: 13.8		Rt: 9.1		Rt: 18.0		Rt: 19.7			
	m/z: 401		m/z: 417		m/z: 701		m/z: 713		m/z: 715		m/z: 715		m/z: 727		m/z: 729		m/z: 729		m/z: 731.2		m/z: 743		m/z: 757			
	Occ. (%)	Mean ng	Occ. (%)	Mean ng	Occ. (%)	Mean ng	Occ. (%)	Mean ng	Occ. (%)	Mean ng	Occ. (%)	Mean ng	Occ. (%)	Mean ng	Occ. (%)	Mean ng	Occ. (%)	Mean ng	Occ. (%)	Mean ng	Occ. (%)	Mean ng	Occ. (%)	Mean ng		
1	100	52.4	0	0	75	115	75	172.6	100	318	0	0	100	248.2	100	695.1	100	134	100	257	0	0	100	723.9	10.8	2715.9
2	100	82.5	40	4	60	60	80	122.3	100	221	50	46.3	100	207.9	100	393.9	100	184	100	435	30	12.8	100	813.7	11.6	2582.7
3	100	111	90	35.4	100	168	100	424.4	100	715	90	122	100	527	100	1018	100	478	100	1051	80	40.2	100	1776	12.0	6464.8
4	100	107	0	0	90	106	100	380	100	717	70	70.1	100	508.1	100	916.6	100	342	100	581	90	55.1	100	1786	11.5	5568.4
5	100	55.9	10	2.1	100	94.4	100	249.3	100	481	100	58.6	100	275.4	100	699.4	100	338	100	549	80	20.6	100	1307	11.6	4130.5
6	100	66.8	0	0	66.7	21.2	66.7	213.5	100	226	33.3	9	100	82.6	100	216	100	171	100	166	0	0	100	742.6	9.3	1914.6
7	100	133	10	3.4	80	57.1	80	150.2	100	341	60	43.8	100	222.2	90	539.3	90	89.5	90	520	70	24.5	100	1131	11.0	3253.9
8	100	47.3	0	0	80	28	80	118.3	100	224	40	7.5	100	109.7	100	222.9	100	125	100	472	0	0	100	667.5	9.6	2022.5
9	100	105	30	6.6	100	304	100	576.1	100	1192	100	253	100	621.2	100	1238	100	737	100	1331	100	151.8	100	2723	11.4	9239.1
10	100	83	55.6	31.7	100	248	100	235.4	100	665	66.7	70.8	100	598.7	100	899.6	100	512	100	905	55.6	32.9	100	1499	11.9	5780.5
11	100	110	80	42.2	100	119	100	464.4	100	959	100	124	100	708.5	100	1377	100	613	100	1209	100	67.3	100	2278	11.9	8072.3
12	100	49.5	0	0	70	124	90	191.3	100	469	70	51.9	100	288.3	100	583.5	100	295	100	672	50	18.3	100	1102	10.9	3844.9
13	100	103	10	0.7	90	103	100	236.4	100	449	70	48.4	100	287.8	100	574.4	100	275	100	539	60	22.2	100	1310	11.8	3949.3
14	100	93.5	10	18.1	40	72	60	58.2	90	208	50	13.4	90	182.7	100	436.2	80	165	80	339	10	2.4	100	683.9	10.2	2272.5
15	100	110	0	0	90	82.3	90	139.8	100	361	80	43	90	161.2	100	393.2	100	230	100	475	40	9.1	100	977.2	10.8	2981.3
16	100	78.4	0	0	88.9	32.4	88.9	78	100	265	66.7	31.4	100	136.9	100	317.2	100	168	100	374	11.1	2	100	709.3	10.0	2192.6

**Fig. 1:**
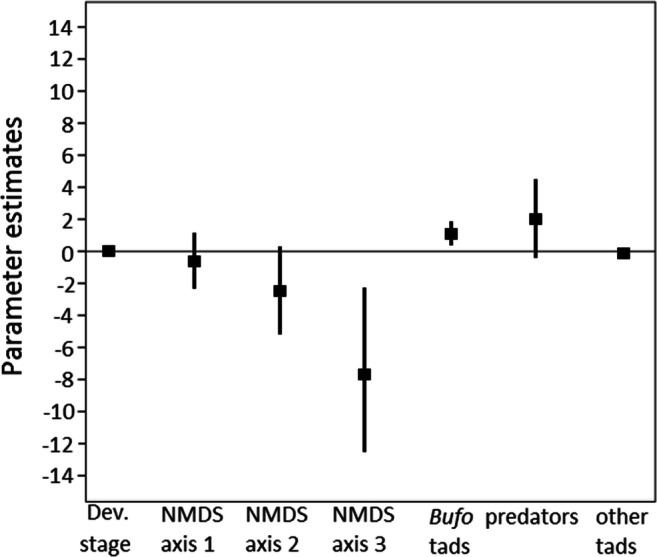
Parameter estimates (black squares) for explanatory variables obtained from the best fitting model describing the pattern of number of bufadienolide compounds (NBC) found in common toad tadpoles in the 16 studied ponds. Vertical lines depict 95% confidence intervals. Abbreviations: *Dev. Stage* developmental stage, *NMDS* nonmetric multi-dimensional scaling, *tads* tadpoles

The best-fitting model describing among-population variation in NBC (range of population mean NBC: 9.33–12 compounds / tadpole, see Table 2) included bacterial community structure, biotic parameters and developmental stage of toad tadpoles as explanatory variables. This model was clearly more supported by the data than the second best model or the null model (ΔAICc = 4.22 and 5.13, respectively; Table OR 2a in Electronic supplementary material2). Further, the 95% confidence intervals computed for the parameter estimate of the NMDS 3 axis and for the density of conspecific larvae did not include zero (see Table OR 3a in Electronic supplementary material 2; Fig. 1), indicating that the number of bufadienolide compounds present in toad tadpoles covaried with some aspects of bacterial community structure, and that the number of compounds increased in parallel with an increasing density of conspecifics. Parameter estimate of the NMDS 2 was also relatively large, but its 95% CI included zero, so that we consider its relationship with NBC to be non-significant (Table OR 3a in Electronic supplementary material 2; Fig. 1).

**Fig. 2:**
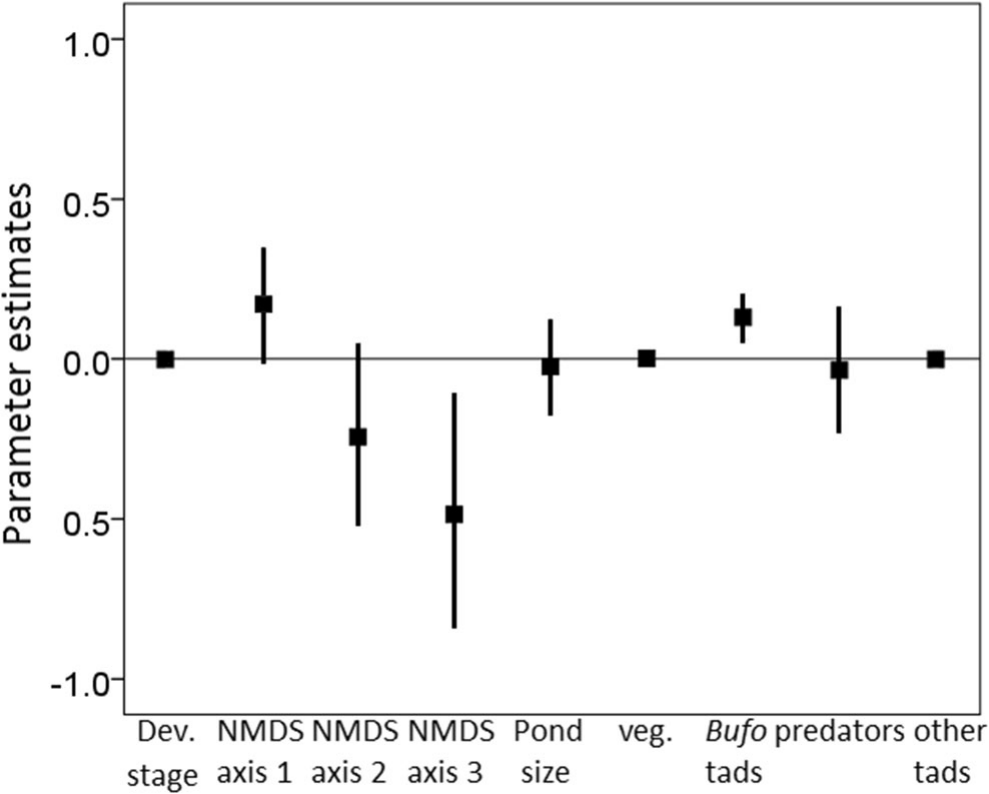
Parameter estimates (black squares) for explanatory variables obtained from model averaging procedures describing the pattern of total bufadienolide quantity (TBQ) found in common toad tadpoles in the 16 studied ponds. Vertical lines depict 95% confidence intervals. Abbreviations: *Dev. Stage* developmental stage, *NMDS* nonmetric multi-dimensional scaling, *veg.* macro-vegetation cover, *tads* tadpoles

Total bufadienolide quantity varied widely among ponds (range of population mean TBQ: 1915–9239 ng / tadpole, see Table 2). The model containing bacterial community data and biotic factors with developmental stages as explanatory variables, followed by the full model containing all predictors were the best-supported models: however, they differed by 3.64 AICc values from each other, and more than eight AICc values from the third best model (Table OR 2b in Electronic supplementary material 2). The 95% confidence intervals for the model-averaged parameter estimates of NMDS 1 axis and the density of conspecific larvae did not include zero, indicating that TBQ was related to bacterial community structure in the aquatic environment and was in a positive relationship with the density of conspecific larvae (Table OR 3b in Electronic supplementary material 2; Fig. 2). Model selection procedures including all measured pond parameters (regardless of correlations between them), and mass corrected TBQ values gave very similar results (Electronic supplementary material 2).

## Discussion

Our results are the first to suggest that toxin content of tadpoles is related to the bacterial community structure of their environment. Both NBC and TBQ were correlated with one or two NMDS axes describing the bacterial community of larval habitats. This may be a cause-effect relationship, because bufadienolides can have antipathogenic and antiparasitic effects (Barnhart et al. 2017; Cunha Filho et al. 2005; Tempone et al. 2008), and Bufonid toads lack AMP-s (Conlon 2011; König et al. 2015), therefore the involvement of bufadienolides in skin-based immune-defense is probable, and their synthesis may be adjusted to the presence or absence of particular pathogens or specific members of bacterial communities. The presence of certain bacteria and changes in the natural microbiota can induce responses in chemical defenses (up- or down-regulation of AMP synthesis) in adult frogs (Mangoni et al. 2001; Miele et al. 1998; Simmaco et al. 1998). Nonetheless, the correlation between toxin content and individual NMDS axes does not inform us about which bacterial taxa are responsible for this relationship because NMDS axes describing bacterial community structure are derived from the visualization of the TRFLP data matrix for the sake of dimension reduction. Physical and chemical parameters were highly variable among habitats (Electronic supplementary material 1), contributing to a distinct bacterial community in each one of the studied ponds (Electronic supplementary material 3, Fig. 3). Therefore, local adaptation to the local microbiota at the level of chemical defenses may contribute to the observed variance in toxicity of common toad tadpoles. Although we did not find a direct effect of abiotic environmental factors on toxin content, these factors can also influence skin associated chemical defenses (Krynak et al. 2015, 2016), thus we cannot completely exclude the possibility that the relationship between bacterial community structure and toxin content resulted from correlations with a non-measured background variable. For example, anthropogenic pollution can influence both toxin content (Bókony et al. 2019; Zhou et al. 2019) and microbial community (Aguinaga et al. 2018; Widenfalk et al. 2008). Finally, some bacteria inhabiting the skin of toads are able to transform bufadienolide compounds (Hayes et al. 2009b; Kamalakkannan et al. 2017), which may have contributed to the observed relationship between NBC and bacterial community structure in our study. This mechanism, however, is unlikely to explain the observed patterns in TBQ.

**Fig. 3:**
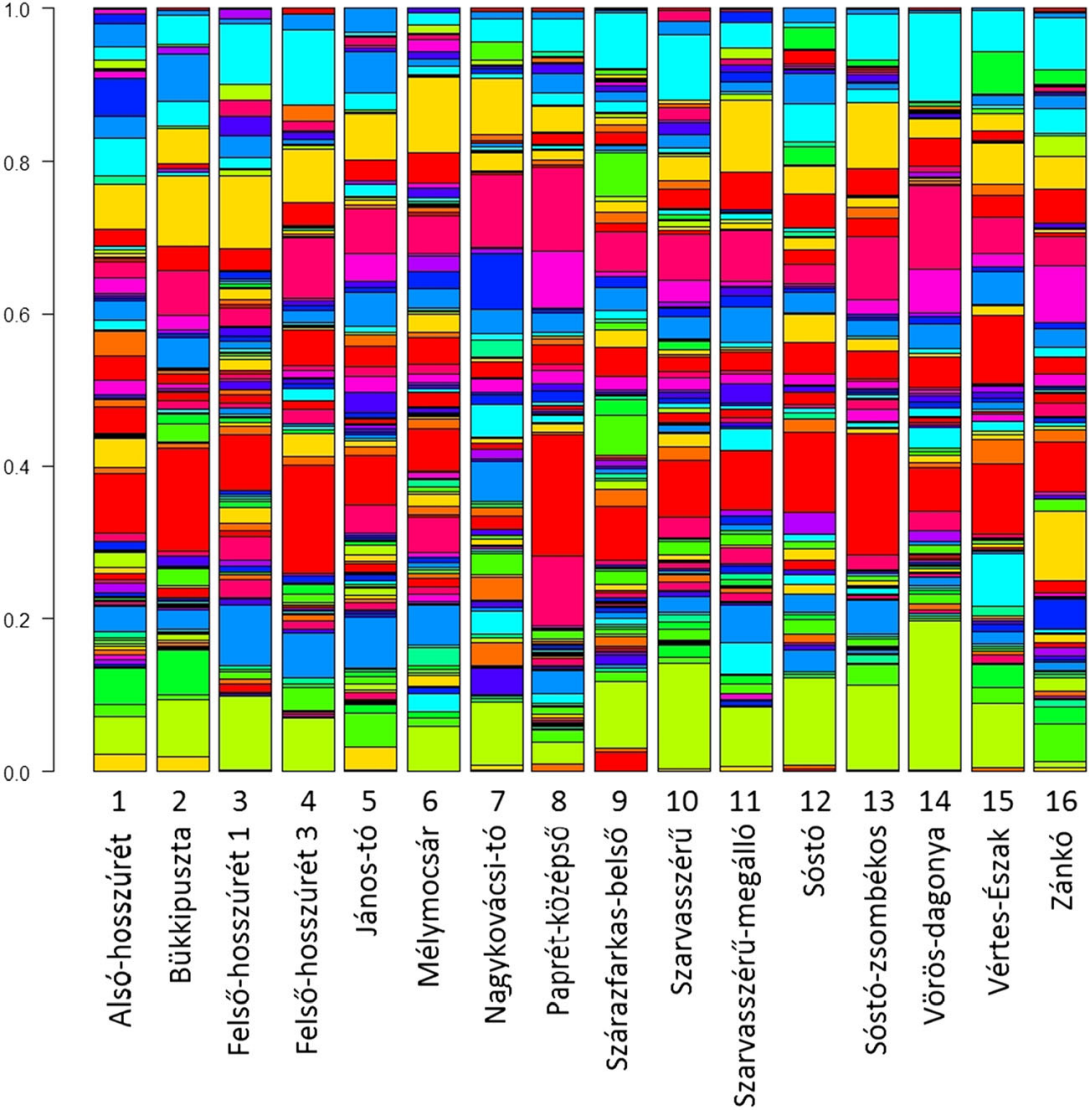
Bacterial community structure in the studied ponds visualized from results of TRFLP. Height of each rectangle in the columns refers to the abundance of the given fragment in the sample. Isochromatic rectangles close to each other between the samples indicate the same fragments. Note that colour palette is vertically repeating, because of the high number of fragments. Numbers are in accordance with the Pond ID column in Table 1

The positive relationship between both NBC and TBQ and the density of conspecific tadpoles is in line with results of a previous correlative study (Bókony et al. 2016) that was conducted one year earlier on a partly overlapping suite of ponds (10 ponds overlapped between the former and the current study). The similarity between results suggests that the observed pattern may be generalized over time and populations, at least within the study area, and suggests that the synthesis of bufadienolides is boosted in response to elevated conspecific density. Experimental studies confirmed these results (Bókony et al. 2018) in our study species. Similarly, AMP synthesis in leopard frogs (*Lithobates pipiens*) was increased after metamorphosis in individuals that had faced strong competition during the larval stage (Groner et al. 2014). These changes in chemical defenses may be interpreted as adaptive plasticity manifesting in the form of responsive immune defense, because the chance of pathogen transmission grows if the density of similar hosts increases (Briggs et al. 2010), which is likely to render enhanced investment into immune-defenses beneficial at high conspecific densities. Presence of predators did not influence skin toxin production, most probably because fishes, which excite the strongest antipredatory responses in chemical defenses (Hettyey et al. 2019), are absent from the sampled ponds, and because the weaker effects of invertebrate predators were masked by those of widely varying densities of conspecific tadpoles.

We observed large variation in tadpoles’ developmental stages among the studied ponds (Table 1). Even though previous laboratory-based experiments showed that toxin quantity of *B. bufo* tadpoles can vary according to developmental stage (Ujszegi et al. 2017; Üveges et al. 2017), we could not avoid such differences among ponds, because natural habitats inherently vary in environmental factors affecting tadpole development (McDiarmid and Altig 1999). However, this variation in developmental stage did not have an effect either on NBC or on TBQ in the present study (Figs. 1 and 2) and the models containing only developmental stage were weakly supported (Table OR 2 in Electronic supplementary material 2). This was likely caused by the fact that the majority of the analyzed tadpoles reached, or passed the middle of their larval development, when toxin production already reached a plateau and does not change much until metamorphosis (Ujszegi et al. 2017; Üveges et al. 2017).

In summary, by investigating correlations between bacterial community structure of aquatic habitats and skin-based chemical defenses of toad tadpoles we demonstrated that chemical defenses of tadpoles are related to the bacterial community structure of their natural aquatic habitats. Furthermore, in accordance with previous findings (Bókony et al. 2016), the toxin content of larval toads was also related to the density of conspecific tadpoles. Revealing the most important bacterial groups that are related to temporal or spatial variation in skin-borne chemical defenses of tadpoles, and perhaps induce these changes, would be an important step towards understanding the processes shaping interactions between environmental microbiota and amphibian chemical defenses.

## Electronic supplementary material

ESM 1 (XLSX 12 kb)

ESM 2 (DOC 52 kb)

ESM 3 (XLSX 55 kb)
