# Supplementary material for: Relationships Between Chemical Defenses of Common Toad (Bufo bufo) Tadpoles and Bacterial Community Structure of their Natural Aquatic Habitat
Source: J Chem Ecol. 2020 May 28;46(5):534–43. doi: 10.1007/s10886-020-01184-4 (PMC7332479; doi:10.1007/s10886-020-01184-4)
Supplement: Supplementary file 2 — (DOC 52 kb) [file 10886_2020_1184_MOESM2_ESM.doc]

Online Resource to:

Relationships between chemical defenses of common toad (*Bufo bufo*) tadpoles and bacterial community structure of their natural aquatic habitat

in *Journal of Chemical Ecology*

János Ujszegi^1*^, Balázs Vajna^2^, Ágnes M. Móricz^3^, Dániel Krüzselyi^3^, Kristóf Korponai^2^, Gergely Krett^2,4^, Attila Hettyey^1^

*^1^Lendület Evolutionary Ecology Research Group, Plant Protection Institute, Centre for Agricultural Research, Herman Ottó út 15, Budapest, 1022, Hungary*

*^2^Department of Microbiology, Eötvös Loránd University, Pázmány Péter sétány 1/C, Budapest, 1117, Hungary*

*^3^Department of Pathophysiology, Plant Protection Institute, Centre for Agricultural Research, Herman Ottó út 15, Budapest, 1022, Hungary*

*^4^Danube Research Institute, Centre for Ecological Research, Karolina út 29, Budapest, 1113, Hungary*

^*^Corresponding author

e-mail: ujszegi.janos@gmail.com

tel.: +36-1-3918607

fax: +36-1-3918653

ORCID ID: 0000-0002-6030-0772

**Table OR 1.** Non-parametric correlations among habitat characteristics of sampling sites (*N* = 16).

| Correlations | | | | | | | | | | | | | | |
| --- | --- | --- | --- | --- | --- | --- | --- | --- | --- | --- | --- | --- | --- | --- |
|  | | | pond size | water depth | shade | vegetation cover | pH | conductivity | NMDS 1 axis scores | NMDS 2 axis scores | NMDS 3 axis scores | *N* of *Bufo* larvae | *N* of non-*Bufo* larvae | *N* of predators |
| Spearman's rho | size | Correlation Coefficient | 1.000 | 0.395 | -0.114 | -0.055 | 0.091 | 0.453 | 0.094 | 0.144 | 0.197 | 0.241 | 0.282 | -0.043 |
|  |  | Sig. (2-tailed) |  | 0.130 | 0.674 | 0.841 | 0.737 | 0.078 | 0.729 | 0.594 | 0.464 | 0.368 | 0.289 | 0.875 |
|  | depth | Correlation Coefficient | 0.395 | 1.000 | -0.099 | -0.282 | 0.081 | 0.471 | 0.015 | 0.166 | ,788^**^ | 0.075 | -,591^*^ | 0.092 |
|  |  | Sig. (2-tailed) | 0.130 |  | 0.714 | 0.290 | 0.766 | 0.065 | 0.956 | 0.539 | 0.000 | 0.783 | 0.016 | 0.734 |
|  | shade | Correlation Coefficient | -0.114 | -0.099 | 1.000 | -0.258 | 0.012 | 0.127 | -0.308 | ,519^*^ | -0.172 | -0.012 | 0.010 | -0.159 |
|  |  | Sig. (2-tailed) | 0.674 | 0.714 |  | 0.335 | 0.965 | 0.639 | 0.246 | 0.039 | 0.525 | 0.965 | 0.970 | 0.557 |
|  | veg | Correlation Coefficient | -0.055 | -0.282 | -0.258 | 1.000 | 0.055 | 0.069 | 0.282 | -0.337 | -0.182 | 0.214 | 0.062 | 0.434 |
|  |  | Sig. (2-tailed) | 0.841 | 0.290 | 0.335 |  | 0.841 | 0.798 | 0.289 | 0.202 | 0.500 | 0.425 | 0.819 | 0.093 |
|  | pH | Correlation Coefficient | 0.091 | 0.081 | 0.012 | 0.055 | 1.000 | ,521^*^ | 0.259 | ,518^*^ | 0.044 | 0.259 | -0.106 | -0.174 |
|  |  | Sig. (2-tailed) | 0.737 | 0.766 | 0.965 | 0.841 |  | 0.039 | 0.333 | 0.040 | 0.871 | 0.333 | 0.696 | 0.519 |
|  | conductivity | Correlation Coefficient | 0.453 | 0.471 | 0.127 | 0.069 | ,521^*^ | 1.000 | 0.024 | ,547^*^ | 0.368 | 0.482 | -0.218 | 0.061 |
|  |  | Sig. (2-tailed) | 0.078 | 0.065 | 0.639 | 0.798 | 0.039 |  | 0.931 | 0.028 | 0.161 | 0.058 | 0.418 | 0.824 |
|  | nmds1 | Correlation Coefficient | 0.094 | 0.015 | -0.308 | 0.282 | 0.259 | 0.024 | 1.000 | -0.012 | -0.021 | 0.200 | -0.018 | 0.099 |
|  |  | Sig. (2-tailed) | 0.729 | 0.956 | 0.246 | 0.289 | 0.333 | 0.931 |  | 0.966 | 0.940 | 0.458 | 0.948 | 0.716 |
|  | nmds2 | Correlation Coefficient | 0.144 | 0.166 | ,519^*^ | -0.337 | ,518^*^ | ,547^*^ | -0.012 | 1.000 | 0.018 | 0.268 | -0.074 | -0.193 |
|  |  | Sig. (2-tailed) | 0.594 | 0.539 | 0.039 | 0.202 | 0.040 | 0.028 | 0.966 |  | 0.948 | 0.316 | 0.787 | 0.473 |
|  | nmds3 | Correlation Coefficient | 0.197 | ,788^**^ | -0.172 | -0.182 | 0.044 | 0.368 | -0.021 | 0.018 | 1.000 | -0.279 | -0.456 | 0.276 |
|  |  | Sig. (2-tailed) | 0.464 | 0.000 | 0.525 | 0.500 | 0.871 | 0.161 | 0.940 | 0.948 |  | 0.295 | 0.076 | 0.301 |
|  | *N* of *Bufo* larvae | Correlation Coefficient | 0.241 | 0.075 | -0.012 | 0.214 | 0.259 | 0.482 | 0.200 | 0.268 | -0.279 | 1.000 | -0.015 | -0.170 |
|  |  | Sig. (2-tailed) | 0.368 | 0.783 | 0.965 | 0.425 | 0.333 | 0.058 | 0.458 | 0.316 | 0.295 |  | 0.957 | 0.530 |
|  | *N* of non-*Bufo* larvae | Correlation Coefficient | 0.282 | -,591^*^ | 0.010 | 0.062 | -0.106 | -0.218 | -0.018 | -0.074 | -0.456 | -0.015 | 1.000 | 0.044 |
|  |  | Sig. (2-tailed) | 0.289 | 0.016 | 0.970 | 0.819 | 0.696 | 0.418 | 0.948 | 0.787 | 0.076 | 0.957 |  | 0.871 |
|  | *N* of predators | Correlation Coefficient | -0.043 | 0.092 | -0.159 | 0.434 | -0.174 | 0.061 | 0.099 | -0.193 | 0.276 | -0.170 | 0.044 | 1.000 |
|  |  | Sig. (2-tailed) | 0.875 | 0.734 | 0.557 | 0.093 | 0.519 | 0.824 | 0.716 | 0.473 | 0.301 | 0.530 | 0.871 |  |
| ** Correlation is significant at the 0.01 level (2-tailed). | | | | | | | | | | | | | | |
| * Correlation is significant at the 0.05 level (2-tailed). | | | | | | | | | | | | | | |

**Table OR 2.** Cumulative link mixed models (a) and linear mixed-effects models (b) ranked by Akaike’s information criterion corrected for sample size (AICc). The number of estimated parameters (K), the AICc difference from the model with the lowest AICc (∆AICc), and the Akaike weight (ω) shown for each model. Predictors were developmental stage of toad tadpoles (DEV), NMDS axes describing bacterial community (BACT), biotic factors (BIOT), abiotic pond parameters (ABIOT). The null model contained only pond ID as a random effect (NULL).

| Predictors | K | AICc | ΔAICc | ω |
| --- | --- | --- | --- | --- |
| a) Number of bufadienolide compunds | |  |  |  |
| DEV + BACT + BIOT | 10 | 296.88 | 0.00 | 0.79 |
| DEV + BACT + BIOT + ABIOT | 12 | 301.10 | 4.22 | 0.10 |
| NULL | 3 | 302.01 | 5.13 | 0.06 |
| DEV + BACT | 7 | 303.68 | 6.81 | 0.03 |
| DEV | 4 | 304.12 | 7.25 | 0.02 |
| DEV + BACT + ABIOT | 9 | 306.17 | 9.30 | 0.01 |
| b) Total bufadienolide quantity |  |  |  |  |
| DEV + BACT + BIOT | 10 | 11.47 | 0.00 | 0.84 |
| DEV + BACT + BIOT + ABIOT | 12 | 15.11 | 3.64 | 0.14 |
| DEV + BACT | 7 | 19.71 | 8.24 | 0.01 |
| NULL | 3 | 22.27 | 10.08 | 0.00 |
| DEV + BACT + ABIOT | 9 | 23.23 | 11.76 | 0.00 |
| DEV | 4 | 24.39 | 12.92 | 0.00 |

**Table OR 3.** Parameter estimates (*b*) and model-averaged parameter estimates (*b*_av_) with 95 % confidence intervals (CI).

| Predictors |  |  | 95 % CI | |  |
| --- | --- | --- | --- | --- | --- |
|  |  |  | lower | upper |  |
| a) Number of bufadienolide compounds | | *b* |  |  |  |
| developmental stage | | 0.0625 | -0.1064 | 0.2314 |  |
| Bacterial community structure | NMDS1 | -0.6831 | -2.3803 | 1.0142 |  |
|  | NMDS2 | -2.5641 | -5.3021 | 0.1739 |  |
|  | NMDS3 | -7.6632 | -12.9204 | -2.4061 | * |
| Biotic characters | [*N* of *Bufo* larvae] | 1.1325 | 0.4290 | 1.8359 | * |
|  | [*N* of predators] | 2.0709 | -0.3466 | 4.4885 |  |
|  | *N* of non-*Bufo* larvae | -0.1473 | -0.3094 | 0.0148 |  |
| b) Total bufadienolide quantity | | *b*_av_ |  |  |  |
| developmental stage | | -0.0019 | -0.0168 | 0.0129 |  |
| Bacterial community structure | NMDS1 | 0.1707 | -0.0017 | 0.3431 |  |
|  | NMDS2 | -0.2429 | -0.5239 | 0.0382 |  |
|  | NMDS3 | -0.4859 | -0.8457 | -0.1261 | * |
| Abiotic characters | [pond size] | -0.0239 | -0.1662 | 0.1185 |  |
|  | vegetation cover | 0.0012 | -0.0019 | 0.0043 |  |
| Biotic characters | [*N* of *Bufo* larvae] | 0.1299 | 0.0641 | 0.1958 | * |
|  | [*N* of predators] | -0.0354 | -0.2237 | 0.1530 |  |
|  | *N* of non-*Bufo* larvae | -0.0017 | -0.0156 | 0.0122 |  |
| Variables in [brackets] were log_10_-transformed before analysis; parameter estimates refer to the transformed values. Asterisks mark CI-s where 0 is not included. | | | | |  |

**Model selection in case of TBQ using all measured pond parameters**

The model averaging process including all measured habitat parameters gave qualitatively very similar results compared to the models in the main text including fewer parameters due to the exclusion of correlated physical parameters (see Table OR 1). We used the log_10_-transformed values of TBQ to ensure normality of model residuals and homogeneity of variances and performed analyses using linear mixed modelling procedures (LMM) comparing fit using Akaike’s information criterion corrected for sample sizes (AICc; for further information please see the Materials and Methods section in the text).

Two models arose as best supported models (difference = 3.49 AICc): the full model, and the model including bacterial and biotic characteristics as well as developmental stage. The other models differed from them with AICc more than 11.73 (Table OR 4). Based on the model-averaged coefficients, the NMDS 3 axis and the density of conspecific larvae had a strong effect on TBQ (Table OR 5).

**Table OR 4.** Linear mixed-effects models ranked by Akaike’s information criterion corrected for sample size (AICc). The number of estimated parameters (K), the AICc difference from the model with the lowest AICc (∆AICc), and the Akaike weight (ω) shown for each model. Predictor variables: developmental stage (DEV), bacterial community (BACT), biotic pond parameters (BIOT), abiotic pond parameters (ABIOT), null model with random effect only (NULL).

| Predictors |  | K | AICc | ΔAICc | ω |
| --- | --- | --- | --- | --- | --- |
| DEV + BACT + BIOT + ABIOT | | 16 | 7.99 | 0.00 | 0.85 |
| DEV + BACT + BIOT | | 10 | 11.47 | 3.49 | 0.15 |
| DEV + BACT |  | 7 | 19.71 | 11.73 | 0.00 |
| DEV + BACT + ABIOT | | 13 | 22.26 | 14.28 | 0.00 |
| NULL |  | 3 | 22.27 | 14.29 | 0.00 |
| DEV |  | 4 | 24.39 | 16.41 | 0.00 |

**Table OR 5.** Model-averaged parameter estimates (*b*_av_) with 95 % confidence intervals (CI) for TBQ in case of inclusion of all measured pond parameters.

| Predictors |  | *b*_av_ | 95 % CI | |  |
| --- | --- | --- | --- | --- | --- |
|  |  |  | lower | upper |  |
| developmental stage | | -0.0020 | -0.0153 | 0.0113 |  |
| Microbial community structure | NMDS1 | 0.1542 | -0.1045 | 0.4128 |  |
|  | NMDS2 | -0.3287 | -0.7340 | 0.0767 |  |
|  | NMDS3 | -0.7054 | -1.2129 | -0.1978 | * |
| Abiotic characters | [pond size] | -0.0784 | -0.3221 | 0.1653 |  |
|  | vegetation cover | 0.0023 | -0.0015 | 0.0061 |  |
|  | shade | 0.0022 | -0.0012 | 0.0056 |  |
|  | [conductivity] | 0.0154 | -0.2524 | 0.2831 |  |
|  | water depth | 0.0043 | -0.0013 | 0.0100 |  |
|  | pH | 0.0355 | -0.1948 | 0.2658 |  |
| Biotic characters | [*N* of *Bufo* larvae] | 0.1116 | 0.0337 | 0.1895 | * |
|  | [*N* of predators] | -0.1118 | -0.3706 | 0.1469 |  |
|  | *N* of non-*Bufo* larvae | 0.0038 | -0.0191 | 0.0267 |  |
| Variables in [brackets] were log_10_-transformed before analysis; parameter estimates refer to the transformed values. Asterisks mark CI-s where 0 is not included. | | | | |  |

**Mass-corrected total bufadienolide quantity (mcTBQ)**

We also calculated mass corrected total bufadienolide quantity (mcTBQ) for each individual by dividing total bufadienolide quantity (TBQ) by tadpole dry mass. We used the log_10_-transformed values of mcTBQ to ensure normality of model residuals and homogeneity of variances and performed analyses using linear mixed modelling procedures (LMM) comparing fit using Akaike’s information criterion corrected for sample sizes (AICc; for further information please see the Materials and Methods section in the text).

Three models arose as the best supported models (maximum difference = 3.36 AICc): the full model, the model including bacterial and biotic characteristics and the model containing only bacterial characteristics and developmental stage. The other models differed from these with AICc larger than 5.36 (Table OR 6). Based on the model-averaged coefficients, two NMDS axes had powerful effects on mcTBQ. Furthermore, developmental stage had a strong negative, whereas vegetation cover among abiotic characteristics a considerable positive effect on mcTBQ. Presence of *Bufo* larvae among biotic characteristics had a marginal effect on mcTBQ (Table OR 7).

The three best supported models contained bacterial community structure, which indicated that this was the most important variable: biotic characteristics were included twice, while abiotic characteristics were included in only one model among the best supported ones. Also developmental stage had a strong effect on mcTBQ (but not on TBQ) based on the results of model averaging procedures, but the model containing developmental stage only was the least supported model (ΔAICc: 10.46) indicating that variation in toxicity was not related to developmental stage alone (Table OR 6).

**Table OR 6.** Linear mixed-effects models ranked by Akaike’s information criterion corrected for sample size (AICc). The number of estimated parameters (K), the AICc difference from the model with the lowest AICc (∆AICc), and the Akaike weight (ω) shown for each model. Predictor variables: developmental stage (DEV), bacterial community (BACT), biotic pond parameters (BIOT), abiotic pond parameters (ABIOT), null model with random effect only (NULL).

| Predictors |  | K | AICc | ΔAICc | ω |
| --- | --- | --- | --- | --- | --- |
| DEV + BACT + BIOT + ABIOT | | 12 | 44.61 | 0.00 | 0.53 |
| DEV + BACT + BIOT | | 10 | 45.83 | 1.22 | 0.29 |
| DEV + BACT |  | 7 | 47.77 | 3.16 | 0.11 |
| DEV + BACT + ABIOT | | 9 | 48.92 | 4.31 | 0.06 |
| NULL |  | 3 | 53.47 | 8.86 | 0.01 |
| DEV |  | 4 | 54.87 | 10.27 | 0.00 |

**Table OR 7.** Model-averaged parameter estimates (*b*_av_) with 95 % confidence intervals (CI) for mcTBQ.

| Predictors |  | *b*_av_ | 95 % CI | |  |
| --- | --- | --- | --- | --- | --- |
|  |  |  | lower | upper |  |
| developmental stage | | -0.0350 | -0.0542 | -0.0158 | * |
| Microbial community structure | NMDS1 | -0.0725 | -0.2934 | 0.1483 |  |
|  | NMDS2 | -0.4625 | -0.8214 | -0.1035 | * |
|  | NMDS3 | -0.8275 | -1.2479 | -0.4072 | * |
| Abiotic characters | [pond size] | 0.0690 | -0.0737 | 0.2118 |  |
|  | vegetation cover | 0.0034 | 0.0003 | 0.0065 | * |
| Biotic characters | [*N* of *Bufo* larvae] | 0.0751 | -0.0068 | 0.1571 |  |
|  | [*N* of predators] | -0.0306 | -0.2802 | 0.2190 |  |
|  | *N* of non-*Bufo* larvae | -0.0121 | -0.0282 | 0.0041 |  |
| Variables in [brackets] were log_10_-transformed before analysis; parameter estimates refer to the transformed values. Asterisks mark CI-s where 0 is not included. | | | | |  |
